# *N*-Alkylated dinitrones from isosorbide as cross-linkers for unsaturated bio-based polyesters

**DOI:** 10.3762/bjoc.10.88

**Published:** 2014-04-22

**Authors:** Oliver Goerz, Helmut Ritter

**Affiliations:** 1 Institut für Organische und Makromolekulare Chemie, Lehrstuhl für Präparative Polymerchemie, Heinrich-Heine-Universität Düsseldorf, Universitätsstr. 1, 40225 Düsseldorf, Germany

**Keywords:** bio-based polyesters, cross-linkers, dinitrones, 1,3-dipolar cycloaddition, isosorbide

## Abstract

Isosorbide was esterified with acryloyl chloride and crotonic acid yielding isosorbide diacrylate (**9a**) and isosorbide dicrotonate (**9b**), which were reacted with benzaldehyde oxime in the presence of zinc(II) iodide and boron triflouride etherate as catalysts to obtain *N*-alkylated dinitrones **10a/b**. Poly(isosorbide itaconite -*co*- succinate) **13** as a bio-based unsaturated polyester was cross-linked by a 1,3-dipolar cycloaddition with the received dinitrones **10a/b**. The 1,3-dipolar cycloaddition led to a strong change of the mechanical properties which were investigated by rheological measurements. Nitrones derived from methyl acrylate (**3a**) and methyl crotonate (**3b**) were used as model systems and reacted with dimethyl itaconate to further characterize the 1,3-dipolaric cycloaddition.

## Introduction

Nitrones represent a class of compounds with a versatile use as electron spin traps [1] and in cycloaddition reactions [2]. As nitrones undergo 1,3-dipolar cycloadditions under mild conditions with a variety of unsaturated substances with a catalyst [3–4] or without a catalyst [5] they are important for the synthetic accessibility of five-membered heterocycles. Efforts have also been undertaken to employ nitrones in the realm of polymer chemistry. For instance, our former reports describe the cycloaddition of bis(*N*-methylnitrone)s and bis(*N*-phenylmaleimide)s , which yielded linear polymers bearing isoxazolidine units with high thermal stability [6]. The synthesis of polymers bearing nitrones as photosensitive material [7–8] was also described. We focused on the preparation of polynitrones as efficient cross-linkers for unsaturated polyesters based on fumaric und maleic acid [9]. In the light of the growing interest in polymers from renewable resources the synthesis of bio-based nitrones as novel cross-linkers for unsaturated bio-polyesters [10] offers an interesting access to biocompatible materials.

This paper presents the synthesis of bio-based *N*-alkylated dinitrones based on isosorbide and the subsequent use of these dinitrones as cross-linkers in a 1,3-dipolar cycloaddition with unsaturated bio-based polyester poly(isosorbide itaconate -*co*- succinate).

## Results and Discussion

**Scheme 1:**
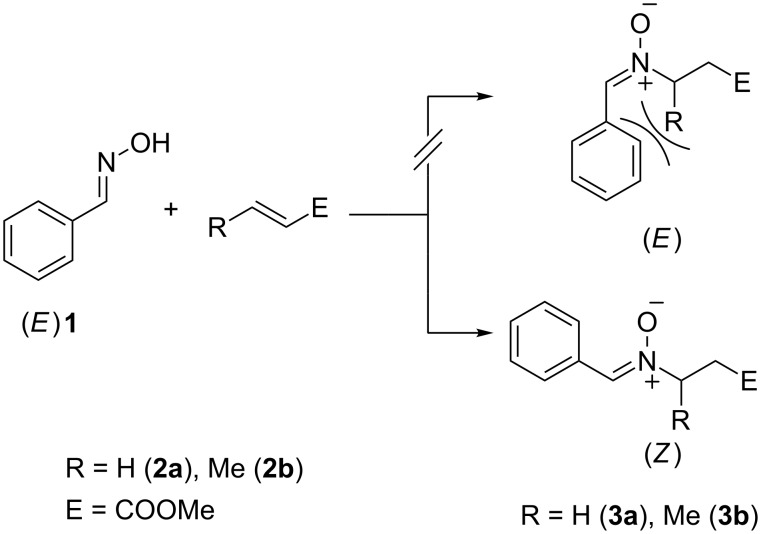
Synthesis of *Z*-configurated model compounds **3a/b** from *E*-benzaldoxime and acrylates **2a/b**.

N-Alkylated dinitrones derived from acrylates and crotonates were prepared and evaluated in respect to their 1,3-cycloaddition with itaconic acid containing polyester resins. To study the course of this cycloaddition the two low molecular model nitrones methyl 3-[benzylidene(oxido)amino]propanoate (**3a**) and methyl 3-[benzylidene(oxido)amino]butanoate (**3b**), were prepared from methyl acrylate (**2a**) and methyl crotonate (**2b**) with *E*-benzaldoxime (**1**) (Scheme 1), respectively.

**Figure 1:**
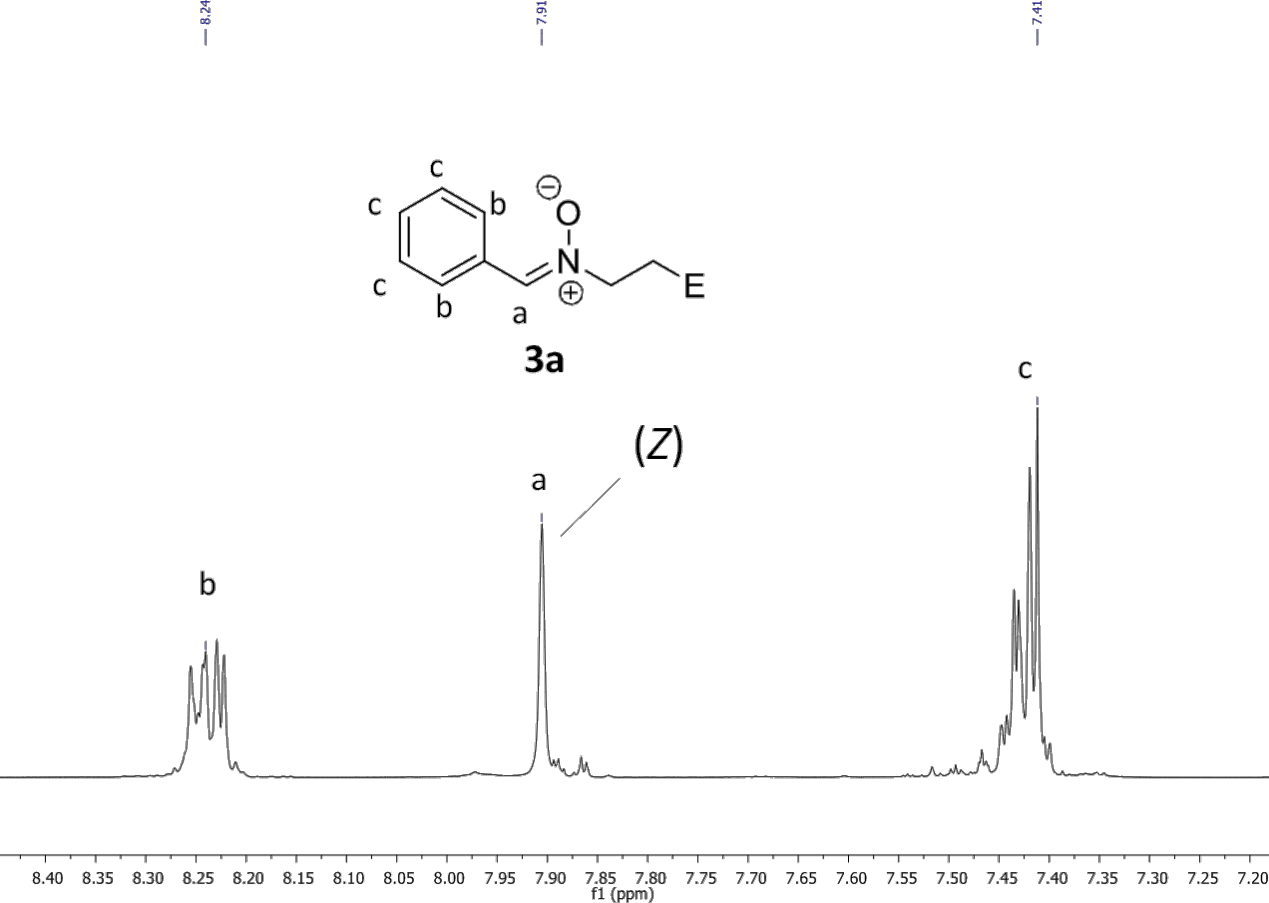
^1^H NMR spectra of **3a** (300 MHz, DMSO-*d*_6_).

The success of the reaction of compounds **3a/b** can be easily verified by ^1^H NMR spectroscopy (Figure 1). Former multiplets of acrylic functionalities in regions of 5.50 ppm to 7.00 ppm vanished after the addition of benzaldoxime. Instead, the nitron signal can be found around 7.90 ppm. The formation of the *Z*-configuration of **3a/b** is evident from the signal at 7.90 ppm. With the formation of the nitrone with an *E*-configuration the proton of the nitron group is exposed to a strong deshielding effect of the *N*-oxide group, which causes a movement of the signal to higher ppm values [11]. The appearance of the *Z*-isomer can also be explained with steric interactions between the phenyl group and the methylene hydrogen atoms of **3a** and the methyl group of **3b**, respectively. Minor impurities in the spectra result from benzaldoxime. FTIR spectroscopy shows the N–O stretch vibration at 1149 cm^−1^ (**3a**) and 1147 cm^−1^ (**3b**) thereby hinting at the formation of nitrones.

In contrast to previous reports [12], we lowered the catalyst amount of ZnI_2_/BF_3_∙OEt_2_ to reduce several side reactions. For example, it could be determined, that high amounts of catalyst led to the oxidation of the oxime to benzaldehyde or the cleavage of nitrones.

**Scheme 2:**
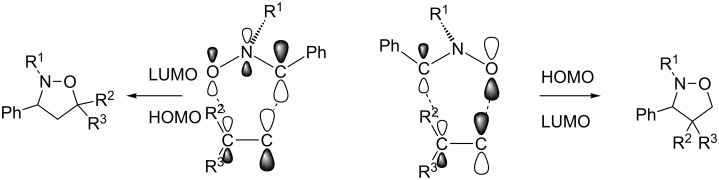
Proposed molecular interactions of nitron **3a** and dimethyl itaconate (**4**).

We expected that the cycloaddition of **3a** with **4** yields two diastereomers for each 4- and 5-substituted isoxazolidines, resulting in four isomers. The proposed molecular interactions are shown in Scheme 2.

**Scheme 3:**
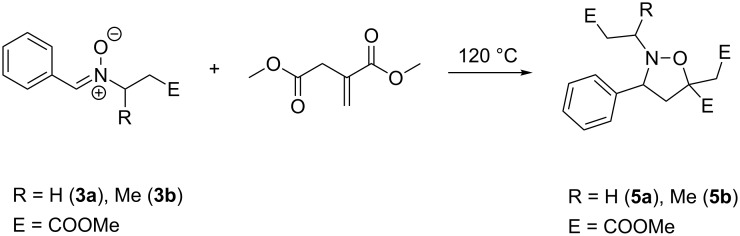
1,3-Dipolaric cycloaddition of nitrones **3a/b** with dimethyl itaconate (**4**).

The model compounds **3a/b** were reacted with an excess of dimethyl itaconate (**4**) obtaining isoxazolidines **5a/b** (Scheme 3).

From the NMR spectra it could be determined that only the 5-substituted isoxazolidine was formed with a ratio of 60/40 of *Z/E*. Although 1,3-dipolar cycloadditions of nitrones with alkenes are controlled by molecular orbital interactions, the steric character of the molecular structures plays a predominant role. In the case of the formation of the 4-substituted isoxazolidine the phenyl group of **3a** and both esters of **4** are expected to approach each other. This causes an electronic repulsion of the sterically demanding groups. Thus, the formation of the 5-substituted isoxazolidine is favored due to reduced steric interactions of the involved groups. Although **3b** shows a slightly different molecular structure the reaction with **4** resulted only in the formation of the 5-substituted isoxazolidine with a diastereomeric ratio of 30/30/20/20.

**Scheme 4:**
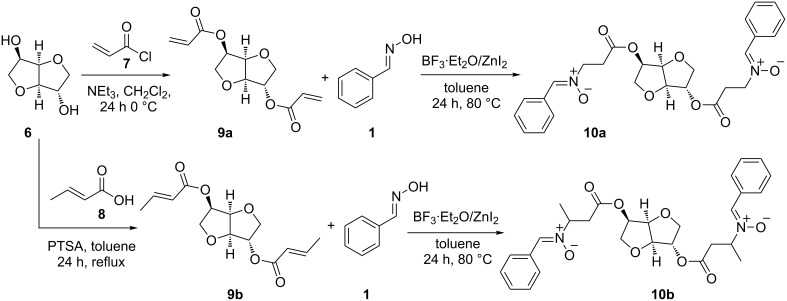
Synthetic route to bio-based dinitrones derived from isosorbide.

To apply the results described above to bio-based cross-linkers, isosorbide (**6**) was esterified with acryloyl chloride (**7**) yielding acrylate **9a**. Dicrotonate **9b** was prepared from isosorbide (**6**) and crotonic acid (**8**, Scheme 4). The obtained compounds **9a/b** were transferred into the corresponding dinitrones **10a/b** with 4 equivalents of benzaldoxime yielding 95% **10a** and 60% **10b**, respectively. In contrast to **3a/b**, the produced dinitrones lacked the solubility in toluene and precipitated during the reaction, resulting in a viscous mass comprised of product, byproducts and catalyst. For purification the crude products were washed with 2-propanol to dissolve byproducts and catalyst. The pure compounds were obtained by additionally washing with diethyl ether.

**Scheme 5:**
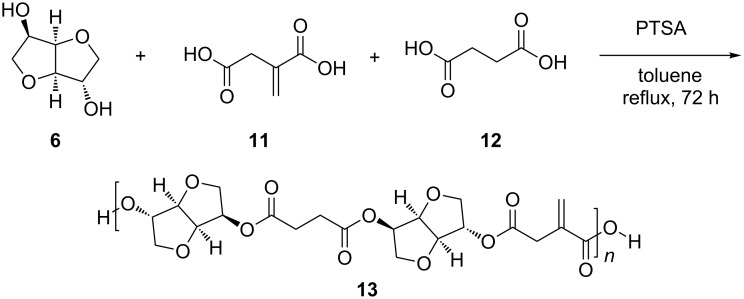
Synthesis of poly(isosorbide itaconate -*co*- succinate) **13** from isosorbide (**6**), itaconic acid (**11**) and succinic acid (**12**).

**Figure 2:**
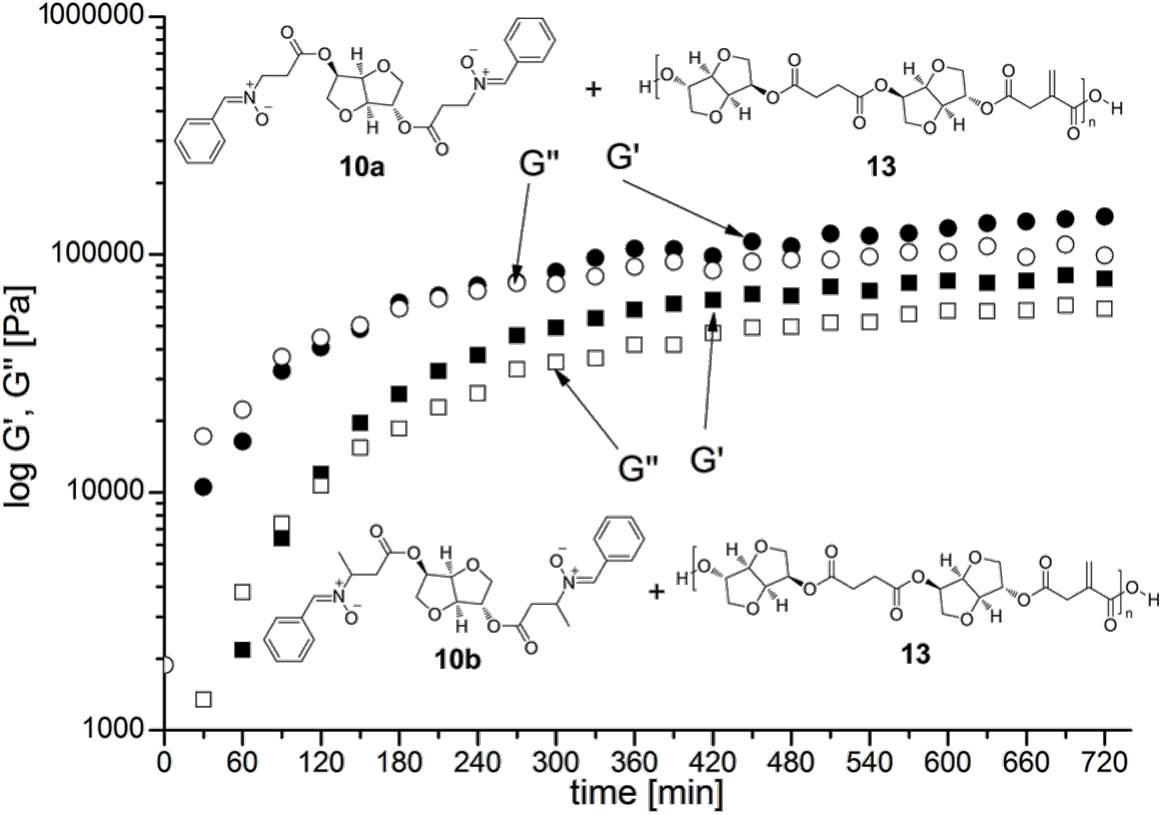
Oscillation measurements during thermal cross-linking of unsaturated polyester **13** with dinitrones **10a** and **10b** at 120 °C.

In order to proof the ability of dinitrones to form three-dimensional networks with itaconate moieties containing polyesters, the bio-based unsaturated polyester **13** was synthesized from isosorbide, itaconic acid and succinic acid under acid catalyzed conditions (Scheme 5). The obtained unsaturated polyester **13** showed 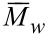 values of 3400 g/mol and a glass transition temperature of 64 °C. The cross-linking of **10a/b** with **13** was investigated by rheological measurements in oscillatory mode. The storage modulus *G'* and loss modulus *G''* which were recorded at a constant shear rate of 1 s^−1^ and a shear stress of 15 Pa are illustrated in Figure 2.

A rapid increase of the *G'* and *G''* values was observed for **10a/b** in the first 120 minutes. Thus, during this period most of the network points were formed. The ongoing cross-linking is accompanied by restrictions in the mobility of the polymer chains and the cross-linkers, which results in lowered reaction rates with flattening values for *G'* and *G''*. Sample **10b** shows a gel point at about 100 minutes, whereas the gel point of **10a** shows up after 210 minutes of heating. This observation can be explained with the different solubilities of **10a** and **10b** in polyester **13**. The molten mixture of dinitrone **10a** and **13** showed a slight turbidity due to phase separation. This means that a certain amount of unsaturated functionalities of the polyester can only be accessed by nitrones after a long reaction time. On the other hand, **10b** was completely soluble in polyester **13**, giving a clear solution without any phase separation. Although the reactivity was reduced, higher values for *G'* and *G''* could be obtained due to the more rigid structure of **10a**.

## Conclusion

In the present work, bio-based dinitrones were prepared from acrylated and crotonated isosorbide with benzaldoxime. They were employed as cross-linkers for unsaturated bio-based polyesters from isosorbide, itaconic acid and succinic acid. The thermal cross-linking could be followed by oscillatory measurements. 1,3-dipolar cycloadditions of model nitrones with dimethyl itaconate confirmed the proposed mechanism of cross-linking.

## Experimental

### Materials

Acryloyl chloride (97%), crotonic acid (98%), itaconic acid (IA, 97%), methyl acrylate (99%), methyl crotonate (98%), succinic acid (SA, 99%), *p*-toluenesulfonic acid, phenothiazine (98%), zinc iodide (98%), and boron trifluoride diethyl etherate (46%) were purchased from Sigma-Aldrich and used as received. Isosorbide (98%) and *E-*benzaldoxime (97%) were purchased from Alfa Aesar, and isosorbide was recrystallized from acetone/ethyl acetate. All used solvents are commercially available and were used without further purification.

#### Measurements

^1^H NMR and ^13^C NMR spectroscopic measurements were performed with a Bruker Avance III spectrometer at 300 MHz and at 75 MHz in either DMSO-*d*_6_ or CDCl_3_ as solvents. Chemical shifts were referenced to the solvent value δ = 2.50 ppm for DMSO-*d*_6_ and δ = 7.26 ppm for CDCl_3_. IR measurements were performed by using a FTIR spectrometer (Nicolet 6700 FTIR) equipped with an ATR unit. Molecular weight distributions were measured by Gel Permeation Chromatography (GPC) with a flow rate of 1 mL/min at room temperature and THF (HPLC Grade, unstabilised – Biosolve) as an eluent. The GPC System compromises a Scharmbeck SFD degasser (Gastor BG12), a FLOW pump (Intelligent PUMP AL-12) and a Scharmbeck SFD (Model S5200) sampler. For detection a Waters 486 Turnable Absorbance Detector and a Schambeck SFD RI 2000 detector were used. A set of columns packed with porous styrene–divinylbenzene–copolymer beads was used for the separation of the analytes (MZ Analysentechnik GmbH, 1 × guard column 100 Å, 3 × columns with 10.000, 1000 und 100 Å). The system was calibrated with polystyrene standards with a molecular range from 575 g/mol to 3,114,000 g/mol. Toluene was added to the analytes as an internal standard. Rheologial measurements were performed by using a Haake Mars II rheometer by ThermoFisher Scientific. For rotation measurements a plate–plate construction (PP35 Ti, *D* = 35; MP35) was used. The temperature was measured with an accuracy of ±0.5 ° C in the measuring plate. The control of the thermostat (Haake DC30 or Haake AC 200-G50) was controlled by the software. Differential scanning calorimetry (DSC) measurements were performed by using a Mettler Toledo DSC 822 controller apparatus in a temperature range between 0 and 200 °C with a heating rate of 10 °C·min^−1^. The glass transition temperature (*T*_g_) values reported are taken from the third heating cycle. EIMS spectra were recorded on a GC–MS integrated system Trace DSG by Thermo Finnigan. The instrument was calibrated in the *m*/*z* range of 1050 Da.

**Synthesis of methyl 3-[benzylidene(oxido)amino]propanoate (3a):** Methyl acrylate (**2a**, 0.86 g, 10 mmol) and benzaldoxime (**1**, 2.42 g, 20 mmol) were placed in a 100 mL round bottom flask and dissolved in 20 mL toluene. Zinc iodide (0.32 g, 1 mmol) and boron triflouride diethyl etherate (0.30 g, 1 mmol) were added. The flask was closed with a plug and the reaction mixture was heated to 80 °C for 24 h. The solvent was evaporated and the crude product dissolved in 20 mL of dichloromethane. Filtration and subsequent purification by column chromatography over silica gel (ethyl acetate/petrol ether 1:1) afforded the pure product in 66% yield. ^1^H NMR (300 MHz, DMSO-*d*_6_) δ (ppm) 8.27–8.21 (m, 2H, Ar-*H*), 7.91 (s, 1H, -C*H*=N-), 7.46–7.39 (m, 3H, Ar-*H*), 4.19 (t, ^3^*J* = 6.4 Hz, 2H, -*CH_2_*-CH_2_-) 3.61 (s, 3H, **-***CH_3_*), 2.92 (t, ^3^*J* = 6.5 Hz, 2H, -CH_2_-*CH_2_*-); ^13^C NMR (125 MHz, DMSO-*d*_6_) δ (ppm) 171 (*C*=O), 134 (-*C*=N-), 131 (Ar-*C*), 130 (Ar-*C*), 128 (4C, Ar-*C*), 61 (-*C*H_2_-CH_2_-), 53 (-O-*C*H_3_), 31 (-*C*H_2_-CH_2_-); FTIR (diamond) 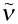 (cm^−1^): 3059, 2996, 2952 (ν, -CH_3_, -CH_2_), 1731 (ν, -C=O), 1172 (ν, -C-O-C-), 1581 (ν, C=N), 1149 (ν, N-O) 753, 691 (ν, -Ar); EIMS *m*/*z*: 221 [M^+^], 205 [M^+^ − C_12_H_15_NO_2_], 190 [M^+^ − C_11_H_13_NO_2_], 132 [M^+^ −C_9_H_10_N], 104 [M^+^ − C_7_H_6_N].

**Synthesis of methyl 3-[benzylidene(oxido)amino]butanoate (3b):** Methyl crotonate (**2b**, 1 g, 10 mmol) and benzaldoxime (**1**, 2.42 g, 20 mmol) were placed in a 100 mL round bottom flask and dissolved in 40 mL toluene. Zinc iodide (0.48 g, 1.5 mmol) and boron triflouride diethyl etherate (0.44 g, 1.5 mmol) were added. The flask was closed with a plug, and the reaction mixture was heated to 80 °C for 24 h. The solvent was evaporated, and the crude product dissolved in 20 mL of dichloromethane. Filtration and subsequent purification by column chromatography over silica gel (ethyl acetate/petrol ether 1:2) afforded the pure product in 46% yield. ^1^H NMR (300 MHz, DMSO-*d*_6_) δ (ppm) 8.28–8.22 (m, 2H, Ar-*H*), 7.95 (s, 1H, -N=C*H*-Ar), 7.44–7.39 (m, 3H, Ar-*H*), 4.60 (m, 1H, -C*H*-CH_3_), 3.57 (s, 3H, -O-C*H_3_*), 3.00 (dd, ^2^*J* = 16.7, ^3^*J* = 8.9 Hz, 1H, -CH-C*H_2_*-), 2.67 (dd, ^2^*J* = 16.7, ^3^*J* = 4.6 Hz, 1H, -CH-C*H_2_*-), 1.37 (d, ^3^*J* = 6.6 Hz, 3H, -CH-C*H_3_*); ^13^C NMR (125 MHz, DMSO-*d*_6_) δ (ppm) 171 (*C*=O), 133 (-*C*=N), 132 (Ar-*C*), 130 (Ar-*C*), 129 (2 C, Ar-*C*), 128 (2 C, Ar-*C*), 67 (-*C*H-CH_3_), 52 (-O-*C*H_3_), 38 (-CH-*C*H_2_-), 19 (-CH-*C*H_3_); FTIR (diamond) 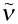 (cm^−1^): 3056, 2986, 2951 (ν, -CH_3_, -CH_2_), 1733 (ν, -C=O), 1175 (ν, -C-O-C-), 1148 (ν, N-O), 753, 691 (ν, -Ar); EIMS *m*/*z*: 207 [M^+^], 192 [M^+^ − C_11_H_13_NO_2_], 134 [M^+^ − C_9_H_12_N], 132 [M^+^ − C_9_H_10_N], 91 [M^+^ − C_7_H_7_].

**General procedure for 1,3-dipolaric cycloaddition of nitrons 3a/b with dimethyl itaconate (4):** A vial was charged with 2 mmol of nitron **3a** or **3b** and 20 mmol of dimethyl itaconate (**4**). The reaction mixture was stirred at 120 °C for 1 h and purified via column chromatography (ethyl acetate/petrol ether 1:4). Yield: 74–75%.

**Methyl 5-(2-methoxy-2-oxoethyl)-2-(3-methoxy-3-oxopropyl)-3-phenylisoxazolidine-5-carboxylate** (**5a): ***cis*-isomer: ^1^H NMR (300 MHz, DMSO-*d*_6_) δ (ppm) 7.39–7.28 (m, 5H, Ar-*H*), 3.92 (m, 1H, Ar-C*H*-), 3.70–3.52 (m, 9H, -O-C*H_3_*), 3.02–2.92 (m, 2H, -C-C*H_2_*-C=O), 2.95 (m, 2H, Ar-CH-C*H_2_*-), 2.89 (m, 2H, -N-C*H_2_*-CH_2_-), 2.48 (m, 2H, -N-CH_2_-C*H_2_*-), 2.42 (m, 2H, Ar-CH-C*H_2_*-); *trans*-isomer: ^1^H NMR (300 MHz, DMSO-*d*_6_) δ (ppm) 7.3–7.28 (m, 5H, Ar-*H*), 3.82 (m, 1H, Ar-C*H*-), 3.70–3.52 (m, 9H, -O-C*H_3_*), 3.02–2.92 (m, 2H, -C-C*H_2_*-C=O), 2.89 (m, 2H, -N-C*H_2_*-CH_2_-), 2.72 (m, 2H, Ar-CH-C*H_2_*-), 2.48 (m, 2H, -N-CH_2_-C*H_2_*-); ^13^C NMR (125 MHz, DMSO-*d*_6_) δ (ppm) 172–169 (-*C*=O), 129–127 (Ar-*C*), 81 (-*C*-), 69 (Ar-*C*H-), 53–51 (-O-*C*H_3_), 48 (Ar-CH-*C*H_2_-), 42 (-C-*C*H_2_-C=O), 41 (-N-*C*H_2_-CH_2_-), 32 (-N-CH_2_-*C*H_2_-); FTIR (diamond) 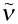 (cm^−1^): 2953, 2917, 2850 (ν, -CH_3_, -CH_2_), 1733 (ν, -C=O), 1195 (ν , N-O), 1173 (ν, -C-O-C-), 756, 701 (ν, -Ar); ESIMS *m*/*z*: 366 [M^+^].

**Methyl 5-(2-methoxy-2-oxoethyl)-2-(4-methoxy-4-oxobut-2-yl)-3-phenylisoxazolidine-5-carboxylate** (**5b): **^1^H NMR (300 MHz, DMSO-*d*_6_) δ (ppm) 7.4–7.25 (m, 5H, Ar-*H*), 4.25–3.80 (m, 1H, Ar-C*H*-), 3.72–3.48 (m, 9H, -O-C*H_3_*), 3.27–2.98 (m, 1H, -N-C*H*-CH_2_-), 3.06–2.84, (m, 2H, -C-C*H_2_*-C=O), 3.02–2.27 (m, 2H, Ar-CH-C*H_2_*-), 2.90–2.13 (m, 2H, -N-CH-C*H_2_*-), 1.04–0.90 (m, 3H, -CH-C*H_3_*-); ^13^C NMR (125 MHz, DMSO-*d*_6_) δ (ppm) 173–169 (-*C*=O), 129–127 (Ar-*C*), 81–80 (-*C*-), 69 (Ar-*C*H-), 56 (-N-*C*H-CH_2_-), 53–51 (-O-*C*H_3_), 48–46 (Ar-CH-*C*H_2_-), 42 (-C-*C*H_2_-C=O), 36 (-N-CH-*C*H_2_-), 20–12 (-CH-*C*H_3_); FTIR (diamond) 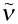 (cm^−1^): 2953, 2920, 2847 (ν, -CH_3_, -CH_2_), 1731 (ν, -C=O), 1197 (ν , N-O), 1170 (ν, -C-O-C-), 757, 701 (ν, -Ar); ESIMS *m*/*z:* 380 [M^+^].

**Synthesis of 2,5-di-*O*-acryloyl-1,4:3,6-dianhydro-D-glucitol (9a):** A 250 mL round bottom flask was charged with isosorbide (7.31 g, 50 mmol) dissolved in 100 mL of dichloromethane. Then triethylamine (11.69 g, 115 mmol) was added and the reaction mixture was cooled to 0 °C. Acryloyl chloride (10.26 g, 110 mmol) was dissolved in 50 mL dichloromethane and added via a dropping funnel under vigorous stirring. The reaction mixture was stirred for additional 24 h after complete addition. Precipitated ammonium chloride was removed by filtration and the obtained solution was washed with 2 × 50 mL of saturated sodium hydrogen carbonate solution. The pure product was received after drying of the organic layer over magnesium sulfate and subsequent removal of the solvent under reduced pressure. Yield: 94%. ^1^H NMR (300 MHz, CDCl_3_) δ (ppm) 6.58–6.33 (m, 2H, *H_2_*C=CH-), 6.22–6.01 (m, 2H, H_2_C=C*H*-), 5.89–5.80 (m, 2H, *H_2_*C=CH-), 5.29–5.16 (m, 2H, -C*H*-O-CO-), 4.87 (t, ^3^*J* = 5 Hz, 1H, -C*H*-CH-), 4.51 (d, ^3^*J* = 4.9 Hz, 1H, -CH-C*H*-), 4.00–3.80 (m, 4H, -CH-C*H_2_*-, -CH-C*H_2_*-); ^13^C NMR (125 MHz, CDCl_3_) δ (ppm) 165 (2C, *C*=O), 132 (2C, H_2_*C*=CH-), 128 (2C, H_2_C=*C*H-), 86 (1C, -*C*H-CH-), 81 (1C, -CH-*C*H-), 78 (1C, -*C*H-O-CO-), 74 (1C, -*C*H-O-CO-), 73 (1C, -CH-*C*H_2_-), 70 (1C, -CH-*C*H_2_-); FTIR (diamond) 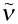 (cm^−1^): 2980, 2876 (ν, -CH_3_, -CH), 1721 (ν, -C=O), 1634 (ν, C=C), 1179 (ν, -C-O-C-); EIMS *m*/*z*: 255 [M^+^], 183 [M^+^ − C_9_H_11_O_4_], 128 [M^+^ − C_6_H_8_O_3_].

**Synthesis of 1,4:3,6-dianhydro-2,5-di-*O*-but-2-enoyl-D-glucitol (9b):** A 250 mL round bottom flask was charged with isosorbide (7.31 g, 50 mmol), crotonic acid (9.04 g, 105 mmol), *p*-toluenesulfonic acid (0.86 g, 5 mmol), phenothiazine (0.02 g, 0.1 mmol) and 80 mL of toluene. The flask was fitted with a Dean–Stark apparatus and the mixture was heated under reflux for 72 h. The solution was washed with 2 × 40 mL of saturated sodium hydrogen carbonate solution and the organic layer was dried over magnesium sulfate. The pure product was obtained by evaporation of the solvent. Yield: 92%. ^1^H NMR (300 MHz, CDCl_3_) δ (ppm) 7.11–6.94 (m, H_3_C-*H*C=CH-), 5.96–5.79 (m, 1H, H_3_C-HC=C*H*-), 5.33–5.08 (m, 2H, -C*H*-O-CO-), 4.86 (t, ^3^*J* = 5 Hz, 1H, -C*H*-CH-), 4.53 (d, ^3^*J* = 4.6 Hz, 1H, -CH-C*H*-), 4.07–3.79 (m, 4H, -CH-C*H_2_*-, -CH-C*H_2_*-), 1.93–1.85 (m, 6H, *H_3_*C-HC=CH-); ^13^C NMR (125 MHz, DMSO-*d*_6_) δ (ppm) δ 165 (2C, *C*=O), 145 (2C, H_3_C-H*C*=CH-), 122 (2C, H_3_C-HC=*C*H-), 86 (1C, -*C*H-CH-), 81 (1C, -CH-*C*H-), 78 (1C, -*C*H-O-CO-), 74 (1C, -*C*H-O-CO-), 74 (1C, -CH-*C*H_2_-), 70 (1C, -CH-*C*H_2_-), 18 (2C, H_3_*C*-HC=CH-); FTIR (diamond) 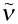 (cm^−1^): 2975, 2876 (ν, -CH_3_, -CH), 1717 (ν, -C=O), 1656 (ν, C=C), 1172 (ν, -C-O-C-); ESIMS *m*/*z*: 283 [M^+^].

**Synthesis of 1,4:3,6-dianhydro-2,5-bis-*O*-{3-[benzylidene(oxido)amino]propanoyl}-D-glucitol (10a):** Compound **9a** (1.27 g, 5 mmol) and benzaldoxime (**1**, 2.42 g, 20 mmol) were placed in a 100 mL round bottom flask and dissolved in 20 mL toluene. Zinc iodide (0.32 g, 1 mmol) and boron triflouride diethyl etherate (0.30 g, 1 mmol) were added. The flask was closed with a plug and the reaction mixture was heated to 80 °C for 24 h. The solvent was evaporated and the crude product washed with 50 mL of isopropanol and 50 mL of diethyl ether. Yield: 95%. ^1^H NMR (300 MHz, DMSO-*d*_6_) δ (ppm) 8.29–8.17 (m, 4H, Ar-*H*), 7.90 (s, 2H, -C*H*=N-), 7.48–7.37 (m, 6H, Ar-*H*), 5.16–5.02 (m, 2H, -C*H*-O-CO-), 4.72 (t, ^3^*J* = 5.3 Hz, 1H, -C*H*-CH-), 4.39 (d, ^3^*J* = 5.3 Hz, 1H, -CH-C*H*-), 4.25–4.13 (m, 4H, O-N-C*H_2_*-CH_2_-), 3.85–3.70 (m, 4H, -CH-C*H_2_*-, -CH-C*H_2_*-), 3.05–2.85 (m, 4H, O-N-CH_2_-C*H_2_*-); ^13^C NMR (125 MHz, DMSO-*d*_6_) δ (ppm) 170 (2C, *C*=O), 134 (2C, -*C*H=N-), 131 (2C, Ar-*C*), 130 (2C, Ar-*C*), 128 (8C, Ar-*C*), 85 (1C, -*C*H-CH-), 80 (1C, -CH-*C*H-), 78 (1C, -*C*H-O-CO-), 74 (1C, -*C*H-O-CO-), 72 (1C, -CH-*C*H_2_-), 70 (1C, -CH-*C*H_2_-), 61 (2C, N-*C*H_2_-CH_2_-), 31 (2C, N-CH_2_-*C*H_2_-); FTIR (diamond) 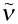 (cm^−1^): 2976, 2871 (ν, -CH_3_, -CH_2_), 1734 (ν, -C=O), 1173 (ν, -C-O-C-), 1148 (ν, N-O), 756, 691 (ν, Ar); ESIMS *m*/*z*: 496 [M^+^].

**Synthesis of 1,4:3,6-dianhydro-2,5-bis-*O*-{3-[benzylidene(oxido)amino]butanoyl}-D-glucitol (10b):** Compound **9b** (1.41 g, 5 mmol) and benzaldoxime (**1**, 2.42 g, 20 mmol) were placed in a 100 mL round bottom flask and dissolved in 20 mL toluene. Zinc iodide (0.48 g, 1.5 mmol) and boron triflouride diethyl etherate (0.44 g, 1.5 mmol) were added. The flask was closed with a plug and the reaction mixture was heated to 80 °C for 24 h. The solvent was evaporated and the crude product washed with 50 mL of isopropanol and 50 mL of diethyl ether. Yield: 60%. ^1^H NMR (300 MHz, DMSO-*d*_6_) δ (ppm) 8.34–8.16 (m, 4H, Ar-*H*), 7.98–7.91 (m, 2H, -C*H*=N-), 7.45–7.37 (m, 6H, Ar-*H*), 5.12–4.93 (m, 2H, -C*H*-O-CO-), 4.69–4.51 (m, 3H, -C*H*-CH-, -C*H*-CH_3_), 4.37–4.22 (m, 1H, -CH-C*H*-), 3.94–3.59 (m, 4H, -CH-C*H_2_*-, -CH-C*H_2_*-), 3.10–2.87 (m, 2H, O-N-CH-C*H_2_*-), 2.75–2.57 (m, 2H, O-N-CH-C*H_2_*-), 1.37 (d, ^3^*J* = 6.5 Hz, 6H, -CH-C*H_3_*); ^13^C NMR (75 MHz, DMSO-*d*_6_) δ (ppm) 170 (2C, *C*=O), 134 (2C, -*C*H=N-), 132 (2C, Ar-*C*), 131 (2C, Ar-*C*), 128 (8C, Ar-*C*), 85 (1C, -*C*H-CH-), 82 (1C, -CH-*C*H-), 78 (1C, -*C*H-O-CO-), 72 (2C, -*C*H-O-CO-, -CH-*C*H_2_-), 71 (1C, -CH-*C*H_2_-), 67 (2C, O-N-*C*H-CH_2_-), 31 (2C, O-N-CH-*C*H_2_-), 19 (2C, -CH-*C*H_3_); FTIR (diamond) 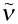 (cm^−1^): 2975, 2936, 2876 (ν, -CH_3_, -CH_2_), 1734 (ν, -C=O), 1580 (ν, C=N), 1176 (ν, -C-O-C-), 1148 (ν, N-O), 755, 692 (ν, Aryl); ESIMS *m*/*z*: 525 [M^+^].

**Synthesis of poly(isosorbide itaconate -*co*- succinate) 13:** A 250 mL round bottom flask was charged with isosorbide (5.85 g, 40 mmol), itaconic acid (2.60 g, 20 mmol), succinic acid (2.36 g, 20 mmol), *p*-toluenesulfonic acid (0.04 g, 0.2 mmol), phenothiazine (0.02 g, 0.1 mmol) and 80 mL toluene. The flask was fitted with a Dean–Stark apparatus and the solution was heated under reflux for 72 h. The solution was concentrated after cooling to room temperature and the crude product was dissolved in a small amount of dichloromethane and precipitated in 300 mL methanol. The product was again dissolved in dichloromethane and the pure polyester was isolated by removing the solvent under reduced pressure. ^1^H NMR (300 MHz, CDCl_3_) δ (ppm) 6.39 (m, 1H), 5.78 (m, 1H), 5.20 (m, 2H), 4.82 (m, 1H), 4.47 (m, 1H), 3.90 (m, 4H), 3.39 (s, 1H), 3.33 (s, 1H), 2.67 (m, 2H); FTIR (diamond) 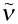 (cm^−1^): 2977, 2935, 2877 (ν, -CH_2_), 1729 (ν, C=O), 1639 (ν, C=C), 1151 (ν, C-O); GPC (THF): 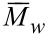 = 3452; *D* = 1.69; DSC: *T*_g_ = 64 °C.
